# Ghent University Hospital’s protocol regarding the procedure concerning euthanasia and psychological suffering

**DOI:** 10.1186/s12910-019-0400-z

**Published:** 2019-09-02

**Authors:** M. Verhofstadt, K. Audenaert, K. Van Assche, S. Sterckx, K. Chambaere

**Affiliations:** 1 End-of-Life Care Research Group, Vrije Universiteit Brussel (VUB) & Ghent University, Corneel Heymanslaan 10, 9000 Ghent, Belgium; 2 Department of Psychiatry and Medical Psychology, Ghent University, Ghent, Belgium; 3 Research Group Personal Rights and Property Rights, Antwerp University, Antwerp, Belgium; 4 Bioethics Institute Ghent, Ghent University, Ghent, Belgium

**Keywords:** Medical assistance in dying, Euthanasia, End-of-life care, Psychiatry, Mental health, Ethics, Hospital guidelines

## Abstract

**Background:**

Notwithstanding fears of overly permissive approaches and related pleas to refuse euthanasia for psychological suffering, some Belgian hospitals have declared that such requests could be admissible. However, some of these hospitals have decided that such requests have to be managed and carried out outside their walls.

**Main text:**

Ghent University Hospital has developed a written policy regarding requests for euthanasia for psychological suffering coming from patients from outside the hospital.

The protocol stipulates several due care criteria that go beyond the requirements of the Belgian Euthanasia Law. For instance, the legally required first and second consulted physicians should all be psychiatrists and be affiliated with a psychiatry department of a Flemish university hospital. Moreover, euthanasia for psychological suffering can only be performed if the advices of these consulted physicians are positive. Importantly, preliminary reflection by the multidisciplinary Hospital Ethics Committee was introduced to discuss every request for euthanasia for psychological suffering coming from outside the hospital.

**Conclusion:**

In this way, the protocol supports psychiatrists faced with the complexities of assessing such requests, improves the quality of euthanasia practice by ensuring transparency and uniformity, and offers patients specialised support and guidance during their euthanasia procedure. Nevertheless, some concerns still remain (e.g. relating to possible unrealistic patient expectations and to the absence of aftercare for the bereaved or for patients whose requests have been refused).

## Background

In Belgium, euthanasia (defined as intentionally terminating life by someone other than the person concerned, at the latter’s request) is decriminalised since 2002 [1]. Euthanasia requests can be carried out on the condition that, inter alia, the patient who is requesting euthanasia is in a medical condition of constant and unbearable physical or psychological suffering that cannot be alleviated and that results from a serious and incurable condition caused by illness or accident, without prospect of improvement [1]. The number of patients euthanased for psychological suffering has steadily increased over the years [2]. In the years 2016–2017, respectively 37 and 40 cases of euthanasia of patients suffering from ‘mental and behavioural disorders’ were reported to the Belgian Federal Control and Evaluation Commission for Euthanasia, amounting to 1.75% of all reported euthanasia cases [3].

As with all other euthanasia requests from patients who are manifestly not expected to die within the foreseeable future, requests based on ‘mental and behavioural disorders’ are subject to two additional procedural requirements as compared to requests from terminally ill patients (i.e. a one-month waiting period between the written euthanasia request and the performance; and the consultation of at least two physicians, including one psychiatrist) [1]. Despite these stricter procedural criteria, heated discussions are taking place, with pleas ranging from prohibiting euthanasia for the mentally ill, over introducing more strict procedural criteria, to extending the scope of the Law (e.g. to patients with advanced dementia or tired of life) [4–7]. In the midst of these debates, several organisations involved in psychiatric care, such as the Belgian branch of the congregation of the Brothers of Charity [8] and the Flemish Association of Psychiatrists [9], have recently published advisory texts on how to more adequately deal with requests for euthanasia for psychological suffering. Interestingly, much earlier, in 2009, an institutional protocol for addressing such requests had already been developed at Ghent University Hospital. This protocol, entitled ‘Procedure Concerning Euthanasia and Psychological Suffering’, is specifically designed to address requests from patients who are referred from outside the hospital. Ghent University Hospital is situated in the Dutch-speaking Northern part of Belgium and has a catchment area of 3.000 patients a day. Its psychiatric unit contains 8 problem-based centres [10].

## Discussion of the protocol

Soon after the Belgian Euthanasia Law came into effect, members of the Hospital Ethics Committee (HEC) informally got in touch with the hospital’s psychiatry department to reflect on the need to introduce a specific procedure for the assessment of requests for euthanasia for psychological suffering. This initiative was prompted by concerns regarding the complexity of assessing the fulfilment of the legal due care criteria, including: (1) the patient’s mental competence, as this might be affected by a psychiatric disorder; (2) the requirement of the incurability of the psychiatric disorder, as some (symptoms of) psychiatric disorders tend to change over time; (3) the requirement of the well-considered nature of the request, as a death wish may be a symptom of a psychiatric disorder; (4) the constant and unbearable nature of the psychological suffering, given that a clear definition and effective assessment instrument are still lacking; and (5) the requirement of the non-alleviability of the psychological suffering. Acknowledging that some patients can make a well-considered euthanasia request on the basis of constant and unbearable psychological suffering that cannot be alleviated by means of therapeutic interventions, it was decided that performing such cases of euthanasia within the hospital’s walls should not be ruled out but needed to be subjected to criteria that are more strict than those prescribed by the Law.

**Fig. 1:**
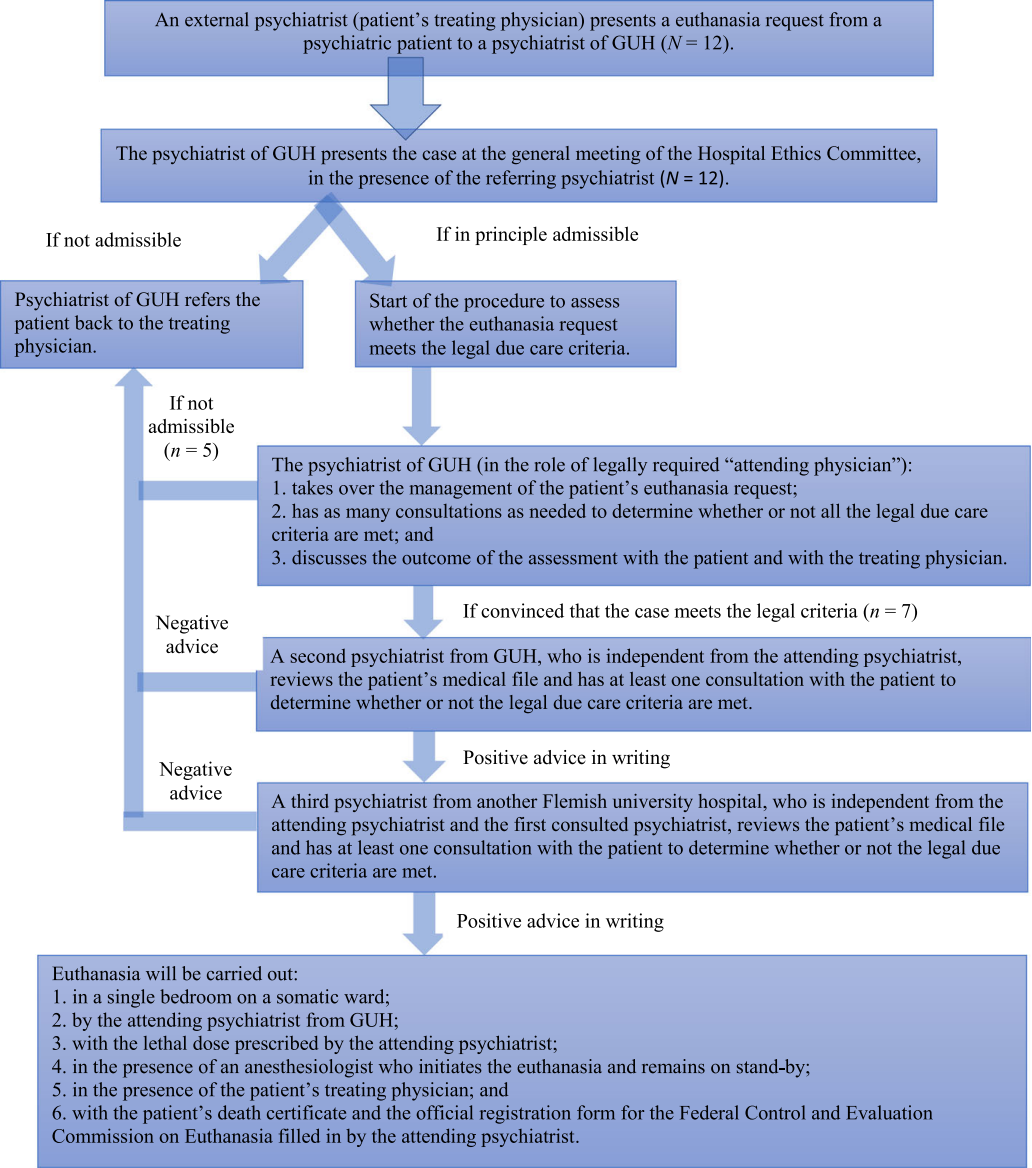
Flow Chart of the Ghent University Hospital (GUH)‘s Protocol Concerning the Procedure regarding Euthanasia and Psychological Suffering

In 2008, a neighbouring psychiatric hospital decided that requests for euthanasia for psychological suffering would need to be managed and carried out outside its walls. When the department of psychiatry of Ghent University Hospital was tentatively sounded out about its preparedness to assess such requests, the HEC agreed on the need to develop a written policy for the management of requests for euthanasia for psychological suffering coming from patients referred by an outside institution or external physician. To develop this policy, the HEC established an ad hoc working group, consisting of some of the permanent HEC members and invited clinical experts. In April 2009, two months after a draft had been debated at a plenary HEC meeting, the final proposal was unanimously approved by the Board of Governors. The step-by-step procedure of this protocol is listed in Fig. 1.

The procedure starts when an external psychiatrist presents a euthanasia request from a psychiatric patient to a psychiatrist of Ghent University Hospital. If the latter is of the opinion that the euthanasia request may be legally admissible, he or she presents the case at a plenary HEC meeting, where the referring psychiatrist also has to be present. Involving the HEC serves to introduce a higher level of scrutiny in the preliminary screening of the eligibility of the euthanasia request. This procedure of shared reflection is an additional safeguard to assist the psychiatrist of Ghent University Hospital in navigating the complexities of the euthanasia request. Moreover, adding this filter protects the hospital’s psychiatry department from being flooded by euthanasia requests from patients coming from outside the hospital who might be attracted by the false prospect of having a straightforward access to euthanasia, once it becomes known that Ghent University Hospital has adopted a policy that is open to such requests.

Although the outcome of the prior discussion is only advisory, in practice it leads to a consensus agreement between all parties. If the HEC declares the case admissible, the assessment of the fulfilment of the legal due care requirements is initiated. If not, the patient is informed about this decision and the case is referred back to the psychiatrist from outside the hospital.

In order to protect the hospital’s psychiatry department from a possible ‘oversupply’ of difficult-to-treat patients (e.g. patients suffering from borderline disorder), discharged by their treating psychiatrists, it is a quintessential premise that the psychiatrist of Ghent University Hospital is involved only in the management of the patient’s euthanasia request, while the patient’s referring psychiatrist remains responsible for the treatment of the patient’s mental disorder(s).

If the case is declared admissible by the HEC, the psychiatrist of Ghent University Hospital assesses the patient’s euthanasia request. At this stage, that psychiatrist takes on the role of the legally required ‘attending physician’ [1]. More specifically, in accordance with the due care criteria provided by the Euthanasia Law, the psychiatrist first has to ascertain: (1) the patient’s competence; (2) that the euthanasia request is voluntary, well-considered, repeated, and not the result of any external pressure; (3) the patient’s constant and unbearable psychological suffering; and (4) that there is no reasonable alternative solution available for the patient’s situation. To that aim, the psychiatrist must have several conversations with the patient spread out over a reasonable period of time, taking into account the progress of the patient’s condition. The patient and her treating psychiatrist are informed about the results of this assessment.

If the psychiatrist of Ghent University Hospital is of the opinion that the euthanasia request meets all the legal due care criteria, a second psychiatrist (i.e. the legally required ‘first consulted physician’) of the hospital’s psychiatry department independently reviews the patient’s medical record and has at least one consultation with the patient in order to evaluate whether or not the legal due care criteria are met. If both psychiatrists give a positive advice, the attending psychiatrist refers the patient for an additional consultation to a psychiatrist (named by the HEC) of a psychiatry department of another Flemish university hospital. In the capacity of legally required ‘second consulted physician’, this third psychiatrist also independently reviews the patient’s medical record and has at least one consultation in order to evaluate whether or not the legal due care criteria are met. Only if all psychiatrists declare the euthanasia request to be in conformity with the legal requirements, euthanasia can be performed at Ghent University Hospital. This procedural requirement goes beyond the legal requirements, as the Euthanasia Law stipulates that the advice of the two consulted physicians is not binding [1].

Figure 1 shows the entire euthanasia procedure with numbers of the requests made (*N* = 12), granted and performed (*n* = 7), or rejected (*n* = 5) at a certain stage of the procedure. Up to the present day, the first psychiatrist always functioned as a gatekeeper who decided which patients were eligible to start the procedure in the Ghent University Hospital protocol.

However, it is important to keep in mind that the protocol stipulates that both the second and the third psychiatrist involved should decide autonomously (e.g. independently from the patient, from the attending psychiatrists and from each other) whether or not to grant the patient’s euthanasia request. In theory, it could occur that some patients who are found eligible by the first psychiatrist are filtered out in the next stages of the procedure. As the first and second psychiatrist are affiliated to the Ghent University Hospital, it is logical that their opinions are crucial in the decision whether or not the psychiatric patient can be euthanased in the hospital. Hypothetically, although this situation has not yet occurred, the opinion of a fourth psychiatrist can be sought when the opinion from the external psychiatrist is negative, if both the first and the second psychiatrist are still of the opinion that the psychiatric patient is eligible for euthanasia. All the opinions of the psychiatrists involved are thoroughly discussed by the HEC at each stage of the procedure, although, in accordance with Belgian law, the advice from the HEC should be considered as providing guidance rather than being legally binding.

According to the protocol of Ghent University Hospital, euthanasia should be performed in a single bedroom in the department of neurology. This is dictated by concern for a negative impact on other patients and staff if euthanasia were to take place in the psychiatry department, possibly giving rise to suicide attempts and ideation or even unleashing a wave of euthanasia requests. It should be noted that, in accordance with the Euthanasia Law, the nurses from the department of neurology have the right to refuse any involvement. In order to protect the patient’s right to privacy, the hospital room where the euthanasia will be performed is booked by a member of the HEC.

The hospital policy prescribes that euthanasia must be performed in the presence of both the patient’s treating and the patient’s attending psychiatrist. Because of the lack of technical expertise on the part of psychiatrists, the euthanasia is initiated by an anaesthesiologist, although the attending psychiatrist is assigned to complete the official registration form and to deliver this document to the Federal Control and Evaluation Commission for Euthanasia.

## Conclusion

During the first 15 years of Belgian euthanasia practice, a few similar hospital-based protocols were developed regarding the assessment of requests for euthanasia for psychological suffering (viz. University Hospital Brussels without and University Hospital Louvain with additional procedural criteria as compared to the Euthanasia Law, e.g. repeated multidisciplinary consultations during the euthanasia procedure) [11]. In its protocol Ghent University Hospital has included criteria that are stricter than legally required: (1) a procedure of preliminary reflection by the HEC; (2) the advices of the legally required first and second consulted physicians should be positive; (3) the consulted physicians should all be experienced psychiatrists affiliated with a psychiatry department of a Flemish university hospital; and (4) the patient’s treating physician should be involved throughout the procedure and be present when the euthanasia is performed. Informal communications received by the authors confirm the positive effects of these additional criteria, especially where it concerns the procedure of preliminary reflection by the HEC. More specifically, discussions with experts from different areas (e.g. other medical specialties, ethics and law) seem to considerably broaden the perspective of the attending psychiatrist, including by making that person more aware of the risk of being (too) susceptible regarding a patient who threatens to commit suicide if the euthanasia request would be refused.

The protocol of Ghent University Hospital has made a valuable contribution to clinical end-of-life practice as: (1) it protects the hospital’s own psychiatrists from an influx of difficult-to-treat patients; (2) it supports its psychiatrists faced with the complexities of assessing a request for euthanasia for psychological suffering; (3) it improves the quality of euthanasia practice by ensuring transparency and uniformity; (4) it offers a way out for psychiatrists working in settings where requests for euthanasia for psychological suffering cannot be met; (5) it offers patients specialised support and guidance during their euthanasia procedure; and, as a consequence, (6) it offers these patients a guarantee that they will not be deprived of therapeutic care. To emphasise the importance of the latter aspect, anecdotal evidence has revealed that some patients felt threatened with involuntary commitment after they expressed a euthanasia request or after mentioning that a euthanasia procedure was initiated, and that some were even excluded from psychiatric stays.

Moreover, the protocol guarantees that the burden of the euthanasia procedure does not fall squarely upon the psychiatrist of Ghent University Hospital who was initially approached. This is achieved via: (1) an a priori reflection by the HEC; (2) the involvement of the patient’s treating psychiatrist; (3) the referral to at least one other independent psychiatrist from the hospital and; (4) to at least one independent psychiatrist from another Flemish university hospital; and (5) the involvement of an anaesthesiologist to administer the lethal dose.

Nevertheless, the protocol also has some shortcomings. For instance, if the euthanasia request is denied, the protocol does not envisage follow-up appointments with the patient and her relatives, but instead just lets the hospital’s physician refer the patient back to her treating physician, who might remain oblivious to the patient’s and her relatives’ needs after refusal in terms of aftercare [12]. Note that this concern also applies for all of the other rejected cases [1].

Even if the euthanasia request is granted, the protocol does not explicitly mention that aftercare should be provided to the bereaved. Moreover, the fact that the psychiatrist who assesses the euthanasia request is not involved in the patient’s treatment could have as an unintended effect that alternatives, such as peer-support recovery-oriented groups, remain underexplored. Furthermore, the protocol may focus too much on assisting and protecting psychiatrists confronted with a request for euthanasia. By contrast, nothing is written on the impact of the euthanasia procedure on the patients. More specifically, the protocol does not include any information on how to anticipate, clarify and address unrealistic patient expectations that the euthanasia request will be granted quasi automatically. For example, if the advices of the first and second psychiatrists are positive, the patient may get the false impression that the consultation of the third psychiatrist is a mere formality.

At a brainstorm meeting with some members of the HEC who were involved in drafting the protocol, it was mentioned that the protocol should be considered as a work in progress that might need to be amended in the light of future challenges. For example, the protocol was written with a specific type of patient in mind, namely that of a severely depressed patient who has become treatment resistant after having been treated in a variety of ambulant and psychiatric in-patient settings for many years, including having been subjected to electroconvulsive therapy without satisfactory results.

In this respect, it should be noted that the protocol does not exclude any psychiatric conditions. However, the protocol was developed at a time when there was a lack of information about which patients would be encountered. In the early years after the adoption of the Euthanasia Law, euthanasia on psychiatric patients was virtually non-existent, as on average only 1 psychiatric patient per year was euthanased. This number increased considerably from 2008 onwards. More detailed information on these cases was published in the biennial report of the Federal Control and Evaluation Committee on Euthanasia in 2010, which is precisely the year in which the protocol was agreed and implemented. Five years after the adoption of the Ghent University Hospital protocol, one quantitative descriptive study (Thienpont et al., 2015) and a recent trend analysis (Dierickx et al., 2017) revealed more details on the characteristics of psychiatric patients in terms of biological sex, age, nature of their psychopathology, and characteristics of the euthanasia procedure and outcome.

Since the Ghent University Hospital developed this protocol before the publication of these studies, it had no information to build on except for their own psychiatrists’ general expertise in psychiatry and personal experience with some cases of euthanasia on psychiatric patients. It turned out that their experiences were congruent with the main findings of these studies. However, these studies describe the most common profiles of patients encountered in practice, whereas other profiles (i.e. in terms of disorder and life context) do occur and can increase the complexity.

In practice, Ghent University Hospital has already been confronted with euthanasia requests made by a variety of patients, including young patients suffering from anorexia nervosa and patients suffering from autism spectrum disorder. Although the characteristics of these cases had not been anticipated, they did not necessitate adjustments to the protocol, as the protocol did not exclude any patient group. The protocol made it clear that psychiatric patients might fulfill all the legal requirements for euthanasia, irrespective of age or nature of the disorder. However, due to their expertise in psychiatry (including in end-of-life care for psychiatric patients), the psychiatrists involved in the development and implementation of the protocol were considered to be the most suitable to adequately manage and discuss these requests and to inform the HEC if there would be a need to make adjustments to the protocol, e.g. to insert additional safeguards.

The HEC also remains alert for the potential situation where future psychiatrists of Ghent University Hospital might have a more permissive stance towards requests for euthanasia for psychological suffering or might want to take a decision without following the procedure outlined in the protocol.

To date, seven cases of euthanasia for psychological suffering have been performed at Ghent University Hospital. The characteristics of these cases cannot be disclosed out of respect for these patients’ and their relatives’ privacy. According to anecdotal evidence provided by members of the HEC, this number is similar to that of other Flemish university hospitals. It is low as compared to the increasing number of cases that are being reported in Belgium, which might suggest that more institutions are prepared to allow euthanasia within their institution. To illustrate this suggestion, the Brothers of Charity recently changed their euthanasia policy so as to allow outside physicians to perform euthanasia on their in-house psychiatric patients [8]. Alternatively, the relatively low number could be due to physicians referring their patients preferably to end-of-life consultation centers that have specialised in complex euthanasia cases and might take a more permissive approach [13]. It should, however, be noted that it is unclear for what reasons some of the external requests might have been refused, since this type of information is not systematically collected.

Moreover, little is known about the number of requests coming from in-house patients, since an institutional protocol that explicitly addresses this issue is still lacking. However, informal communications suggest that such a request has recently been formulated by an in-house psychiatric patient and was discussed by the HEC, which stressed the need to establish a protocol to address this type of requests.

It should be noted that, during a roundtable meeting with the HEC, the HEC emphasised that financial costs or gains should in no way influence access to euthanasia, which should only be based on medical expertise, sound decision-making, interdisciplinary reflection, and transparent communication and responsibility. Therefore, during the assessment procedure no additional costs are charged to the patient except for the normal costs of the consultation of the psychiatrists. If the patient is euthanased, the invoice of that intervention is split into three parts: one part will be borne by the patient’s basic health insurance, the second part will be borne by the patient’s hospitalisation insurance (if applicable), and the third part will need to be borne by the patient. If the patient has no relatives and has not paid in advance, the invoice will only be met after a few years (e.g. from the patient’s estate or, failing that, the patient’s debt might eventually even be paid off by Ghent University Hospital).

In sum, the benefits of holding a preliminary reflection meeting with a multidisciplinary HEC, formulating a clear stance on euthanasia for psychological suffering and providing clear guidance on addressing its challenges in a way that guarantees the correct application of the legal due care criteria and the hospital’s additional criteria, would be important to be taken into account by other jurisdictions that consider medical assistance in dying for psychiatric patients.

## Data Availability

Not applicable.

## Abbreviation

HEC: Hospital Ethics Committee
